# Outcomes of cardiac screening in elite para-football players in the United Kingdom

**DOI:** 10.1136/bjsports-2025-110406

**Published:** 2025-12-12

**Authors:** Kentaro Yamagata, Richard Weiler, Raghav T Bhatia, Jamie S McPhee, Charlotte M Cowie, Sanjay Sharma, Aneil Malhotra

**Affiliations:** 1 Institute of Sport, Manchester Metropolitan University, Manchester, UK; 2 Institute of Sport, Exercise & Health, University College London, London, UK; 3 Hull University Teaching Hospitals NHS Trust, Hull, UK; 4 St George’s University of London, London, UK; 5 The Football Association, St. George's Park, Burton upon Trent, UK

**Keywords:** Athletes, Para-Athletes, Football, Cardiology

## Abstract

**Objectives:**

Studies to date have under-represented cardiac characteristics of para athletes, despite their unique cardiovascular physiology and risks. This study examined the cardiac electrical and structural characteristics and outcomes of pre-participation cardiac screening in elite para-football players.

**Methods:**

Between 2011 and 2024, 156 consecutive para-football players underwent pre-participation evaluation comprising a health questionnaire, 12-lead ECG and echocardiogram. Players with symptoms or abnormal preliminary investigations were evaluated further, including exercise testing and cardiac MRI. Findings were compared with 1000 consecutive sex-matched and ethnicity-matched non-para-football players. Mean follow-up was 4.7±3.1 years.

**Results:**

The mean age of para-football players was 21.2±5.2 years. The majority were male (71.2%) and white ethnicity (84%), followed by mixed-race (7.1%), Asian (4.5%) and Afro-Caribbean (3.8%) ethnicity. Para-football players reported cardiac symptoms more frequently and required more follow-up than non-para players (14.7% vs 6.6%; p=0.001). Para players demonstrated less sinus bradycardia (35.3% vs 44.5%; p=0.037) and a similar prevalence of abnormal T-wave inversion (1.9% vs 3.0%; p=0.622). A short PR interval was observed in 4.5% of para players compared with 0.5% of non-para players (p<0.001), although QTc intervals were similar between the two groups (402±24 ms vs 399±19 ms; p=0.138). Four (2.6%) para-football players received diagnoses associated with sudden cardiac death (SCD) versus three (0.3%) non-para players (p=0.005). Six (3.8%) para-football players were diagnosed with minor cardiovascular conditions versus 1.8% non-para players (p=0.17). In total, 10 (6.4%) para-football players were diagnosed with cardiac pathology versus 21 (2.1%) non-para-football players (p=0.005), all of whom required management or were kept under surveillance. No SCD events occurred over 4.7±3.1 years of follow-up in para-football players.

**Conclusion:**

In this study, para-football players were three times more likely to be diagnosed with a cardiac condition requiring management and/or surveillance compared with non-para players. Moreover, para-football players had a higher prevalence of serious cardiac disease associated with SCD. Cardiac screening inclusive of ECG is warranted in this growing cohort of athletes.

**WHAT IS ALREADY KNOWN ON THIS TOPIC**

- Exercise cardiovascular research has historically focused on non-para athletes, particularly male subjects. Para-football shows greatest participation at elite levels, with blind football played by 46 countries internationally. Para athletes have unique cardiovascular risks but remain understudied despite growing participation in elite sports.

**WHAT THIS STUDY ADDS**

- This study highlights that para-football players were over twice as likely to require further evaluation from cardiac screening and three times more likely to harbour cardiac pathology than non-para-football players.

**HOW THIS STUDY MIGHT AFFECT RESEARCH, PRACTICE OR POLICY**

- This study shows that routine cardiac screening inclusive of a resting ECG is warranted in para athletes, owing to their elevated burden of cardiac pathology.

## Introduction

 Among the various para athletic disciplines, football has observed the greatest participation at elite levels, particularly with the growth of para-football leagues and tournaments worldwide. Para-football players represent a broad range of eligible impairments, including paraplegia, visual or hearing impairment, and cerebral palsy. There may be significant overlap of such conditions with cardiac pathology that could be overlooked.^1^,^4^ Initiatives by sports governing bodies aim to increase participation in para-football and raise the profile and support for national para-football teams.^5^ Blind football alone is one of the most popular sports in the Paralympic Games and is played by 46 countries at the international level.^6^

The cardiac characteristics of para athletes have been under-represented in studies to date, despite the unique cardiovascular risks associated with concomitant autonomic dysfunction and systemic inflammation.^7 8^ Furthermore, individuals with underlying conditions common in para athletes, such as cerebral palsy and spinal cord injury, have been shown to carry a higher burden of cardiovascular disease compared with those without these conditions.^9 10^ These individuals may harbour potentially serious cardiac disease, which in some cases may remain undetected, as lower exercise intensity in certain para athletes can reduce the likelihood of unmasking symptoms. However, many elite para athletes reach training volumes comparable to non-para athletes.^11^ In fact, the England Football Association (FA) initiated a dedicated cardiac screening programme since 2011 for para-football players competing at the international level. This study aims to characterise the cardiac electrical and structural characteristics of para-football players and report the outcomes of a mandatory pre-participation cardiac screening programme, with comparative analysis against a cohort of non-para-football players to identify key differences between the groups.

## Methods

### Screening programme

This was a prospective cohort study of 156 elite para-football players competing at international level undergoing mandatory cardiac screening between May 2011 and August 2024, with retrospective analysis of cardiac screening using contemporary international criteria and comparison to a matched non-para-football player cohort. Written consent for the screening was obtained from each football player or the player’s guardian if younger than 16 years old, in accordance with the FA governance department.

The evaluation comprised a health questionnaire, standard 12-lead ECG and echocardiogram. A high blood pressure (BP) was defined as a systolic BP of ≥140 mm Hg and/or diastolic BP of ≥90 mm Hg at the time of screening. A player was only labelled as having hypertension if this was self-reported as a known diagnosis or that their BP was elevated on re-evaluation after the screening, having been confirmed on repeat measurements with a validated automated device. All cardiac investigations were performed by appropriately accredited sonographers, and the results were reviewed by expert sports cardiologists. The result of the cardiac evaluation for each football player was categorised into either ‘normal’ (no findings requiring action), ‘borderline/uncertain—further testing required’ (findings that are likely physiological or minor but require additional evaluation) or ‘abnormal—further testing required’ (findings that may suggest cardiac pathology and require additional evaluation). The decision to deem football players fit to train and play was made in accordance with (1) a shared decision-making approach and (2) contemporary recommendations.^12 13^

### ECG

A standard 12-lead ECG was performed, with the participant in the supine position during quiet respiration using a GE Marquette Hellige recorder (Milwaukee, Wisconsin, USA) or Philips Pagewriter Trim III (Bothell, Washington, USA) at a paper speed of 25 mm/s and amplification of 0.1 mV/mm. Wave voltages were measured in each lead using callipers and a millimetre ruler as described elsewhere.^14^

Left ventricular (LV) and right ventricular (RV) hypertrophy were defined using the Sokolow–Lyon voltage criteria.^15 16^ T-wave inversion (TWI) was adjudicated per International Criteria, accounting for age and ethnicity: anterior TWI confined to V_1_–V_3_ in athletes <16 years and V_1_–V_4_ in Afro-Caribbean athletes if preceded by ST elevation were considered physiological; inferior and lateral TWI were considered abnormal. A short PR interval was defined as a PR interval of <120 ms. The QT interval was corrected for heart rate using Bazett’s formula.^17^

ECGs were initially analysed by regional cardiologists with expertise in sports cardiology and inherited cardiac conditions. All ECGs were re-evaluated independently by the first author (KY) in 2024 with retrospective application of the International Criteria for Electrocardiographic Interpretation in Athletes (online supplemental table S1).^13^ The first author was initially blinded to any pathological conditions that were subsequently diagnosed with echocardiography. The ECGs of para-football players with equivocal ECG findings were discussed with the principal investigator (AM) who made the final adjudication.

### Echocardiography

Two-dimensional transthoracic echocardiography was performed by sonographers fully accredited by the British Society of Echocardiography in accordance with the standard American Heart Association (AHA) and European Society of Cardiology (ESC) protocols.^18 19^ Sonographers were blind to findings of the health questionnaire and ECG. Standard views were obtained, and wall thickness, cavity dimension measurements, aortic dimensions and identification of the origins of the left and right coronary arteries were made in accordance with established guidelines.^18^,^20^

### Further evaluation

Players with potential cardiac symptoms, suspicious family history, abnormal physical examination and abnormalities on the ECG or echocardiogram were investigated further, including exercise testing, cardiac monitoring and cardiac magnetic resonance (CMR) imaging.^21^ CMR was performed in selected cases with clinical indications such as unexplained ECG and/or echocardiogram findings, suspected cardiomyopathy or myocarditis. CMR was performed using a 3T Siemens MAGNETOM Vida scanner and 3T GE Discovery Scanner. The CMR protocol consisted of initial scout acquisition, followed by long-axis and short-axis cine imaging, phase-contrast imaging, native T1 mapping sequence using MOLLI 5(3)3 acquisition scheme and postcontrast late-gadolinium enhancement imaging. T2 mapping sequence was also incorporated depending on clinical needs. CMR imaging was interpreted according to the Society for Cardiovascular Magnetic Resonance and ESC guidelines.^22 23^

An a priori list of cardiac conditions associated with sudden cardiac death (SCD) included any form of cardiomyopathy, myocarditis, anomalous origin of coronary artery, Wolff–Parkinson–White (WPW) pattern, long QT syndrome, any ventricular arrhythmia, channelopathy, Brugada syndrome, severe degree of valvular pathology and significant aortopathy. Hypertrophic cardiomyopathy, dilated cardiomyopathy (DCM) and arrhythmogenic cardiomyopathy (ACM) were considered in accordance with predetermined criteria and established guidelines.^24^,^30^ The diagnosis of long-QT syndrome was based on a corrected QT interval (QTc) of ≥500 ms or a QTc of 470–490 ms associated with abnormal T-wave morphology, paradoxical prolongation of the QT interval with exercise or a positive genetic test.^31 32^ The WPW ECG pattern was based on findings of a short PR interval and a slurred upstroke to the QRS complex.

Findings from the present study were compared with a cohort of 1000 consecutive sex and ethnicity frequency-matched non-para-football players from the UK, who underwent the same screening protocol under the auspices of the English FA between March and August 2017. Both cohorts underwent the same assessment protocol: after an initial evaluation by an expert sports cardiologist, all investigations were independently reviewed by the first author (KY) and the principal investigator (AM), who were blinded to the outcomes. ECG and echocardiography parameters were also compared between male and female participants.

### Assessment of outcomes

The follow-up period for our study cohort was 4.7±3.1 years. Data on survival status to date among all screened para-football players were obtained from the FA and attending cardiologists, most of whom were part of the expert FA consensus panel.

### Statistical analysis

Statistical analysis was performed using Microsoft Excel V.2408. Normal distribution of all continuous variables was examined using the Shapiro–Wilk test. Differences in proportion between two independent populations were analysed using χ^2^ test or Fisher exact test as appropriate, and continuous variables were analysed using the t-test. Continuous variables are presented as mean±SD.

### Patient and public involvement

Patients and/or the public were not involved in the design, conduct, reporting or dissemination plans of our research.

### Equity, diversity and inclusion

The author group consists of one woman and six men, and consists of junior, mid-career and senior researchers; although all members of the author group practise in one country, the author group has a diverse ethnic background. Our study population included all elite para-football players who had a mandatory cardiovascular screening and included both male and female athletes from different socioeconomic backgrounds participating in elite para-football. However, socioeconomic backgrounds in terms of cardiovascular impact were not assessed.

## Results

### Study population demographics and symptoms

Demographic characteristics of both para and non-para-football players are summarised in table 1. The mean age of the 156 para-football players was 21.2±5.2 years and 111 (71.2%) were male. The cohort comprised 41 (26.3%) blind players, 15 (9.6%) partially sighted players, 72 (46.2%) deaf players and 28 (17.9%) players with cerebral palsy. The majority of para-football players were of white ethnicity (84.0%), followed by mixed-race (7.1%), Asian (4.5%) and Afro-Caribbean ethnicity (3.8%). The mean body surface area (BSA) was 1.90±0.15 m^2^ in male para players and 1.70±0.11 m² in female para players (p<0.001). Para-football players trained for an average of 8.3±3.9 hours per week, while non-para-football players trained for an average of 10.7±1.6 hours per week (p<0.001).

**Table 1:** Demographics of para-football players and non-para-football players

Baseline characteristics	Para-football players (n=156)	Non-para-football players (n=1000)	P value
Age	21.2±5.2	20.5±4.4	0.11
Male players	111 (71.2%)	774 (77.4%)	0.09
Body surface area (m^2^)	1.85±0.16	1.86±0.12	0.45
Ethnicity			
White	131 (84.0%)	798 (79.8%)	0.22
Afro-Caribbean	6 (3.8%)	71 (7.1%)	0.13
Asian	7 (4.5%)	22 (2.2%)	0.09
Mixed-race	11 (7.1%)	97 (9.7%)	0.29
Training hours (per week)	8.3±3.9	10.7±1.6	<0.001

Cardiac symptoms were reported in 34 (21.8%) para players. The most commonly reported symptom was dizziness (13.5%), followed by chest pain (7.1%), loss of consciousness (5.1%) and palpitations (4.5%). Para players reported cardiac-related symptoms at a higher rate compared with non-para players (21.8% vs 4.6%; p<0.001). Also, female para-football players experienced one or more cardiac symptoms over twice as frequently as male para-football players (33.3% vs 17.1%; p=0.045). A family history of premature coronary artery disease or SCD was reported in 3.2% of the para-football players compared with 1.5% among 1000 non-para-football players (p=0.23).

The mean BP among para-football players was 123/72 mm Hg compared with 127/69 mm Hg among non-para-football players. None of the para-football players reported a history of hypertension compared with only one (0.1%) non-para-football player (p=0.69). There was no significant difference between the prevalence of high BP at the time of screening among para-football players and non-para-football players (6.4% vs 4.8%; p=0.51). After re-evaluation, none of the para-football players nor non-para-football players had a new diagnosis of hypertension.

### Electrical characteristics

ECG characteristics of para and non-para-football players are summarised in table 2. There were no differences in physiologic ECG patterns between para players and non-para players except that sinus bradycardia and sinus arrhythmia were less frequent in para-football players (35.3% vs 44.5%; p=0.037 and 7.1% vs 37.2%; p<0.001, respectively) and para players demonstrated a longer PR interval (149 vs 141 ms; p<0.001).

**Table 2:** ECG characteristics of para-football players compared with non-para-football players

ECG parameters	Para-football players (n=156)	Non-para-football players (n=1000)	P value
Sinus bradycardia*	55 (35.3%)	445 (44.5%)	0.037
Sinus arrhythmia	11 (7.1%)	372 (37.2%)	<0.001
LV hypertrophy	29 (18.6%)	240 (24.0%)	0.166
RV hypertrophy	0 (0%)	23 (2.3%)	0.108
Mean PR interval (ms)	149±23	141±18	<0.001
Mean QTc interval (ms)	402±24	399±19	0.138
First-degree atrioventricular block	4 (2.6%)	32 (3.2%)	0.859
Left bundle branch block	0 (0%)	1 (0.1%)	1.0
Incomplete right bundle branch block	7 (4.5%)	80 (8.0%)	0.11
Right bundle branch block†	0 (0%)	4 (0.4%)	0.953
Interventricular conduction delay>140 ms	1 (0.6%)	1 (0.1%)	0.634
Early repolarisation	11 (7.1%)	286 (28.6%)	<0.001
Abnormal T-wave inversion	3 (1.9%)	30 (3.0%)	0.622

* Sinus bradycardia is defined as <60 beats per minute.

† Right bundle branch block only includes complete right bundle branch block with QRS duration ≥120 ms.

LV, left ventricular; QTc, corrected QT; RV, right ventricular.

No significant differences in ECG abnormalities were found between para and non-para players. Abnormal TWI was observed in 3 (1.9%) para players versus 30 (3.0%) non-para players (p=0.622). Specifically, two anterior TWIs in players over 16 years old and white ethnicity, and one inferolateral TWI was present among para players. Examples of ECGs showing TWI are presented in online supplemental figure S1. A short PR interval was identified in seven (4.5%) para-football players versus five (0.5%) non-para players (p<0.001). One para player (0.6%) exhibited a delta wave on the ECG, leading to a diagnosis of WPW pattern. Early repolarisation was less frequently demonstrated in para-football players (7.1% vs 28.6%; p<0.001).

### Structural changes

Echocardiographic parameters of para-football players compared with non-para-football players are summarised in table 3. Para-football players demonstrated smaller end-diastolic diameters for the LV (47.5 vs 51.1 mm; p<0.001) and the RV (33.3 vs 35.9 mm; p<0.001). When indexed with BSA, para-football players also demonstrated a smaller indexed LV mass compared with non-para-football players (78.7 vs 85.4 g/m^2^; p<0.001). Para-football players demonstrated more diastolic dysfunction with lower mitral valve E/A ratio compared with non-para-football players (1.97 vs 2.11; p=0.007). Four (2.6%) para-football players demonstrated aortic root dilatation compared with one (0.1%) non-para-football player (p=0.001). Valvular regurgitation of more than a mild degree of severity was not demonstrated among the cohort of para-football players compared with two (0.2%) non-para-football players (p=1.0). Coronary artery abnormalities were not detected in both para and non-para-football players.

**Table 3:** Echocardiography parameters of para-football players compared with non-para-football players

Echocardiography parameters	Para-football players (n=156)	Non-para-football players (n=1000)	P value
End-diastolic LV diameter (mm)	47.5±4.8	51.1±4.8	<0.001
Indexed end-diastolic LV diameter (mm/m^2^)	26.0±2.4	28.0±2.4	<0.001
Maximal wall thickness (mm)	8.9±1.5	8.9±1.5	1.0
Relative wall thickness (mm)	0.38±0.06	0.36±0.05	<0.001
LV mass (g)	145±45	158±40	<0.001
Indexed LV mass (g/m^2^)	78.7±21.1	85.4±18.6	<0.001
Mitral valve E/A ratio	1.97±0.58	2.11±0.67	0.007
RV D1 (mm)	33.3±5.7	35.9±5.2	<0.001
Aortic root (mm)	27.0±4.3	27.1±3.7	0.784

LV, left ventricular; RV, right ventricular.

### Sex differences in para-football players

A comparison of ECG and echocardiogram data between male and female para-football players is presented in table 4. There was an increased prevalence of sinus bradycardia and LV hypertrophy by Sokolow–Lyon criteria on ECG among male para-football players versus females (42.3% vs 17.8%; p=0.006 and 24.3% vs 4.4%; p=0.008, respectively). The corrected QT interval was longer in female para-football players (422 vs 398 ms; p<0.001).

**Table 4:** Comparison between male and female para-football players

ECG and echocardiography parameters	Male para-football players (n=111)	Female para-football players (n=45)	P value
Sinus bradycardia*	47 (42.3%)	8 (17.8%)	0.006
Sinus arrhythmia	8 (7.2%)	3 (6.7%)	1.0
LV hypertrophy	27 (24.3%)	2 (4.4%)	0.008
Incomplete right bundle branch block	7 (6.3%)	0 (0%)	0.194
Early repolarisation	11 (9.9%)	0 (0%)	0.034
Mean PR interval (ms)	149±23	146±23	0.463
Mean QTc interval (ms)	398±23	422±23	<0.001
First-degree AV block	3 (2.7%)	1 (2.2%)	1.0
Interventricular conduction delay (>140 ms)	1 (0.9%)	0 (0%)	1.0
Abnormal T-wave inversion	1 (0.9%)	2 (4.4%)	0.199
End-diastolic LV diameter (mm)	48.9±4.5	44.6±4.0	<0.001
Indexed end-diastolic LV diameter (mm/m^2^)	26.0±2.37	26.0±2.53	1.0
Maximal wall thickness (mm)	9.4±1.3	8.0±1.3	<0.001
Relative wall thickness	0.38±0.06	0.36±0.06	0.063
LV mass (g)	161±42	113±30	<0.001
Indexed LV mass (g/m^2^)	85.2±20.4	65.6±15.9	<0.001
Mitral valve E/A ratio	2.01±0.58	1.87±0.57	0.170
RV D1 diameter (mm)	34.7±4.5	30.3±4.7	<0.001
Indexed RV D1 diameter (mm/m^2^)	18.4±2.4	17.8±2.6	0.186
Aortic root (mm)	27.8±4.6	25.1±3.0	<0.001

* Sinus bradycardia is defined as<60 beats per minute.AV.

AV, atrioventricular; LV, left ventricular; QTc, corrected QT; RV, right ventricular.

Male para-football players had a larger absolute LV diameter compared with females (48.9 vs 44.6 mm; p<0.001) as well as for the RV (34.7 vs 30.3 mm; p<0.001). However, when indexed for BSA, there were no statistically significant differences between sexes for both LV and RV diameters. Male para-football players also demonstrated a thicker maximal wall thickness (9.4 vs 8.0 mm; p<0.001), as well as an increased LV mass, including LV mass values indexed for BSA (85.2 vs 65.6 g/m^2^; p<0.001).

### Outcomes of the cardiac screening

Para players were more than twice as likely to undergo further investigations compared with non-para players (14.7% vs 6.6%; p=0.001). Following initial cardiac evaluation, 23 para players (14.7%) were referred for further follow-up and/or investigations. Of these, six players (26.1%) were referred due to cardiac-related symptoms and/or a family history of SCD. Eight players (34.8%) with abnormal ECGs and nine players (39.1%) with abnormal echocardiography underwent further evaluation.

Four para-football players were recommended for additional testing due to short PR intervals, with one displaying features of the WPW ECG pattern, who then underwent a curative accessory pathway ablation and returned to play. One para-football player was referred due to an abnormally prolonged QTc on resting ECG>480 ms, which paradoxically increased on standing. Genetic testing revealed a variant of unknown significance. The player was diagnosed with a probable long QT syndrome and was commenced on beta-blocker therapy (nadolol) and returned to play under clinical surveillance.

Three para players were referred for CMR due to a dilated aortic root, while one player with a bicuspid aortic valve also manifested a dilated aortic root and was kept under close clinical surveillance. CMR was additionally performed in three players: one player with a small pericardial effusion in whom CMR was normal; one player with LV hypertrophy on echocardiogram in whom CMR was within normal limits and one player with a background of Duchenne’s muscular dystrophy with reduced biventricular function on echocardiogram, in which CMR confirmed DCM. The player with DCM was advised to refrain from competitive sports and subsequently retired. Three more para players required surveillance for T-wave abnormalities, two of which were in the anterior leads and one in the lateral leads. One player was diagnosed with a small atrial septal defect, necessitating further follow-up.

Among non-para players, three (0.3%) players were diagnosed with cardiac conditions associated with SCD. Two (0.2%) demonstrated evidence of a WPW pattern on the resting ECG and were referred for an electrophysiology study with accessory pathway catheter ablation. One (0.1%) was diagnosed with hypertrophic cardiomyopathy following comprehensive evaluation with exercise testing, CMR and cardiac rhythm monitoring. Other minor cardiac conditions were identified in 18 (1.8%) non-para players, including six (0.6%) players with an atrial septal defect, six (0.6%) players with a bicuspid aortic valve, two (0.2%) players with a patent ductus arteriosus, two (0.2%) players with a valvular regurgitation (> mild degree of severity), one (0.1%) player with a mild dilatation of the aortic root and one (0.1%) player with a small ventricular septal defect.

**Figure 1:**
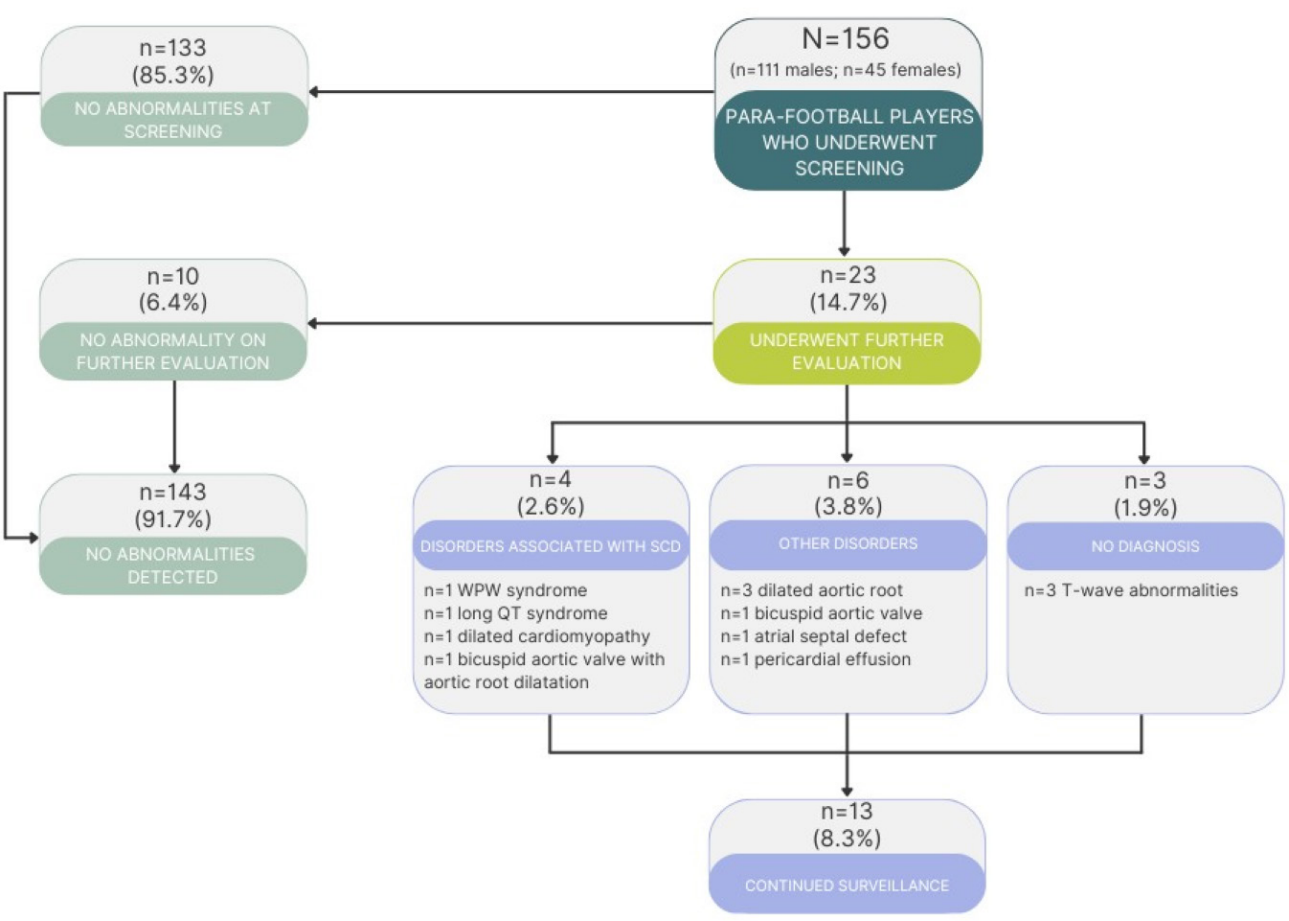
Outcomes of cardiac screening in para-football players. SCD, sudden cardiac death; WPW, Wolff–Parkinson–White.

As summarised in figure 1, cardiac screening led to cardiac diagnoses with elevated risk of SCD in four para players (2.6%; WPW, likely long QT syndrome, bicuspid aortic valve with aortopathy and DCM). Two (1.3%) of these had cerebral palsy and the other two (1.3%) were deaf. The four para-football players with serious pathology were restricted from playing while under investigation. All the para-football players except the DCM case were appropriately treated, counselled and returned to play as part of a shared decision-making process. The prevalence of cardiac conditions associated with SCD among para players was higher compared with non-para players (2.6% vs 0.3%; p=0.005).

Six (3.8%) para players were diagnosed with minor cardiac conditions, including three (1.9%) players with aortic dilatation, one (0.6%) with an atrial septal defect, one (0.6%) with a bicuspid aortic valve and one (0.6%) with a mild chronic pericardial effusion. From these six para players with minor cardiac conditions, four (2.6%) were deaf players, one (0.6%) was a blind player and one (0.6%) was a partially sighted player. The total prevalence of all cardiac conditions among para players warranting management and/or surveillance was 6.4%, which was higher compared with 2.1% among non-para players (p=0.005). A total of 13 para players (8.3%), including three with T-wave abnormalities, remained under surveillance. In comparison, a total of 52 non-para players (5.2%) remained under surveillance, including 29 with T-wave abnormalities without diagnoses, one with left bundle branch block and one with non-specific inter-ventricular conduction delay (p=0.16). During the follow-up period of 4.7±3.1 years, there were no reports of SCD among para-football players who underwent cardiac screening.

## Discussion

While large studies on non-para athletes provide extensive data, the para athlete population remains understudied. Previous research by Pelliccia *et al* has shown that paralympic athletes, particularly those with spinal cord injuries, may face unique cardiovascular challenges due to autonomic dysfunction and impaired preload.^33^ Athletes with non-spinal cord injuries tend to display greater cardiac remodelling, particularly when involved in endurance sports. Our study addresses a critical gap by offering insights into the cardiovascular status of para-football players. We observed that the referral rate for further evaluation was twice as frequent among para athletes than non-para athletes.^21^ The prevalence of diagnosed cardiac disorders among para athletes was also significantly higher compared with non-para athletes, which justifies the need for cardiovascular screening to identify at-risk individuals.

Approximately a quarter of the players in the present study reported symptoms suggestive of potential cardiac issues, with this proportion rising to one-third among female para-football players. Para-football players demonstrated a smaller biventricular size and LV mass, as well as a decreased prevalence of sinus bradycardia, sinus arrhythmia and a relatively longer PR interval compared with non-para-football players. This observation may be a consequence of reduced training loads as we have observed in this study, which could also be limited by underlying health conditions. The prevalence of abnormal TWI was similar among para and non-para-football players, consistent with previous studies, although limited to non-para athletes.^21 34 35^

In terms of sex-specific cardiac findings, male para-football players exhibited a higher prevalence of sinus bradycardia and voltage criteria for LV hypertrophy. In contrast, female para-football players had a longer corrected QT interval compared with their male counterparts. These findings are consistent with previous ECG observations in non-para players.^36 37^ Echocardiographic data from our study showed that female football players had smaller biventricular dimensions compared with males, although when indexed to BSA, there was no difference. The maximal wall thickness and LV mass were greater in male para players, which is similar to non-para player comparisons between sexes.^37^

Current recommendations, such as those from ESC and AHA, are primarily based on data from non-para players.^12 38^ These guidelines may not align with the unique cardiovascular profile of para players, who have demonstrated a higher frequency of follow-ups and investigations, as well as a greater prevalence of cardiac conditions associated with SCD. The higher prevalence of serious disease in para players may be attributed to several factors, including lower earlier detection due to a historically lower frequency of cardiac screening in this cohort, and a lower exercise load to unmask potentially serious cardiac disease. Deafness and blindness may be secondary to syndromic diseases associated with cardiac involvement, including cardiomyopathy and ion channel diseases, and cerebral palsy is linked with obesity and metabolic syndrome.^39 40^ Although not applicable to our study, athletes with spinal cord injury associated with paraparesis may be prone to the cardiovascular complications associated with repetitive systemic inflammation from urinary tract sepsis. Likewise, conduction intervals may be delayed in para athletes with neuromuscular disease. Para athletes with limb deficiencies may demonstrate altered BSA. These findings suggest a more comprehensive approach to cardiac screening in these populations may be necessary.

### Clinical implications

We acknowledge that the routine use of echocardiography together in all para players with normal ECGs and no cardiac symptoms goes beyond current international recommendations.^13^ The FA’s cardiac screening programme is one of the longest-standing in British sport and the most comprehensive of its type by using ECG and echocardiogram which is above the international recommendations.^13^ The programme was initiated over 30 years ago, following the SCD in an academy footballer from ACM. This protocol has remained since and highlights the benefits of a high-resource programme from which such data can be analysed. For younger players aged under 15 years old, the FA conducts ECGs only with echocardiogram if required. We support the international guidelines with a risk-stratified approach for settings with constrained resources with prioritisation of athletes for echocardiogram in those with positive findings from the history, examination or ECG. Particular attention should be taken when assessing para athletes in light of their increased prevalence of cardiac pathology.

### Limitations

Several limitations of our study should be noted. We relied on voluntary reporting of symptoms from para players by a health questionnaire. There is a risk of under-reporting of symptoms, although the frequency was still higher than that of non-para players. Another limitation is that our study cohort was drawn exclusively from elite para-football players competing for England, and the majority of players were of white ethnicity, which may restrict the transferability of findings to other ethnic groups, countries or para-sports. The focus on football may not capture the full spectrum of para athletes, as cardiac load, training intensity and underlying impairments differ in each sport. However, football is a mixed power-endurance discipline and one of the most popular sports worldwide, which may partly enhance the relevance of our findings. We note that our sample is smaller compared with large-scale data published on non-para athletes with a relatively modest follow-up period and included only para-football players of the highest ability. Therefore, our findings may underestimate the burden of cardiac disorders among non-elite players. Nevertheless, this is the first study of its kind with data derived from the largest screening programme for para players in the UK.

## Conclusion

In this study, para-football players report more symptoms and demonstrate a three times higher prevalence of cardiac disease than non-para players of similar age. The prevalence of exercise-related ECG changes is comparable to that observed in non-para players. However, structural adaptations in para players tend to be modest. Similar to non-para players, male para players exhibit greater LV wall thickness and mass compared with female counterparts. Routine cardiac screening appears warranted to detect underlying cardiac conditions in para athletes. Screening protocols tailored to para athletes could ensure that this unique group of athletes receive the optimal cardiac care.

## Supplementary material

10.1136/bjsports-2025-110406 online supplemental file 1

## Data Availability

No data are available.
