# Supplementary material for: Outcomes of cardiac screening in elite para-football players in the United Kingdom
Source: Br J Sports Med. 2025 Dec 12;60(4):e110406. doi: 10.1136/bjsports-2025-110406 (PMC13018733; doi:10.1136/bjsports-2025-110406)
Supplement: online supplemental file 1 [file bjsports-60-4-s001.docx]

**Supplementary Table S1: Comprehensive List and Definitions of ECG Criteria**

| **ECG finding** | **Definition** |
| --- | --- |
| Sinus bradycardia | <60 bpm |
| Sinus arrhythmia | Heart rate variation with respiration: rate increases during inspiration and decreases during expiration |
| Left atrial enlargement | Prolonged P wave duration of >120 ms in leads I or II with negative portion of the P wave ≥1 mm in depth and ≥40 ms in duration in lead V_1_ |
| Right atrial enlargement | P wave ≥2.5 mm in II, III or aVF |
| Ectopic atrial rhythm | P waves are a different morphology compared with the sinus P wave, such as negative P waves in the inferior leads (‘low atrial rhythm’) |
| 1° AV block | PR interval >200ms |
| Mobitz type I (Wenckebach) 2° AV block | PR interval progressively lengthens until there is a non-conducted P wave with no QRS complex; the first PR interval after the dropped beat is shorter than the last conducted PR interval |
| Mobitz type II 2° AV block | Intermittently non-conducted P waves with a fixed PR interval |
| 3° AV block | Complete heart block |
| Ventricular pre-excitation | PR interval <120 ms with a delta wave (slurred upstroke in the QRS complex) and wide QRS (≥120 ms) |
| Junctional escape rhythm | QRS rate is faster than the resting P wave or sinus rate and typically less than 100 beats/min with narrow QRS complex unless the baseline QRS is conducted with aberrancy |
| Left axis deviation | −30° to −90° |
| Right axis deviation | >120° |
| LV + RV hypertrophy | Isolated QRS voltage criteria for left (SV1 + RV5 or RV6 >3.5 mV) or right ventricular hypertrophy (RV1 + SV5 or SV6 >1.1 mV) |
| Premature ventricular contractions | ≥2 premature ventricular contractions per 10 s tracing |
| Pathological Q waves | Q/R ratio ≥0.25 or ≥40 ms in duration in two or more leads (excluding III and aVR) |
| Incomplete RBBB | rSR’ pattern in lead V_1_ and a qRS pattern in lead V_6_ with QRS duration <120 ms |
| Complete RBBB | rSR′ pattern in lead V_1_ and an S wave wider than R wave in lead V_6_ with QRS duration ≥120 ms |
| Complete LBBB | QRS ≥120 ms, predominantly negative QRS complex in lead V_1_ (QS or rS) and upright notched or slurred R wave in leads I and V_6_ |
| Profound non-specific intraventricular conduction delay | Any QRS duration ≥140 ms |
| Epsilon wave | Distinct low amplitude signal (small positive deflection or notch) between the end of the QRS complex and onset of the T wave in leads V_1_-V_3_ |
| ST segment depression | ≥0.5 mm in depth in two or more contiguous leads |
| Early repolarisation | J point elevation, ST elevation, J waves or terminal QRS slurring in the inferior and/or lateral leads |
| Brugada type 1 pattern | Coved pattern: initial ST elevation ≥2 mm (high take-off) with downsloping ST segment elevation followed by a negative symmetric T wave in ≥1 leads in V_1_-V_3_ |
| Prolonged QT interval | QTc ≥470 ms (male) QTc ≥480 ms (female) QTc ≥500 ms (marked QT prolongation) |
| T wave inversion | ≥1 mm in depth in two or more contiguous leads; excludes leads aVR, III and V_1_ |
| Anterior | V_2_-V_4_  excludes: black athletes with J-point elevation and convex ST segment elevation followed by TWI in V_2_-V_4_; athletes < age 16 with TWI in V1-V_3_; and biphasic T waves in only V_3_ |
| Lateral | I and aVL, V_5_ and/or V_6_ (only one lead of TWI required in V_5_ or V_6_) |
| Inferolateral | II and aVF, V_5_-V_6_, I and aVL |
| Inferior | II and aVF |
| AV = atrioventricular; bpm = beats per minute; ECG = electrocardiogram; LBBB = left bundle branch block; LV = left ventricle; RBBB = right bundle branch block; RV = right ventricle; TWI = T wave inversion.  **Source**: Drezner et al. (2017) ^1^ | |
|  | |


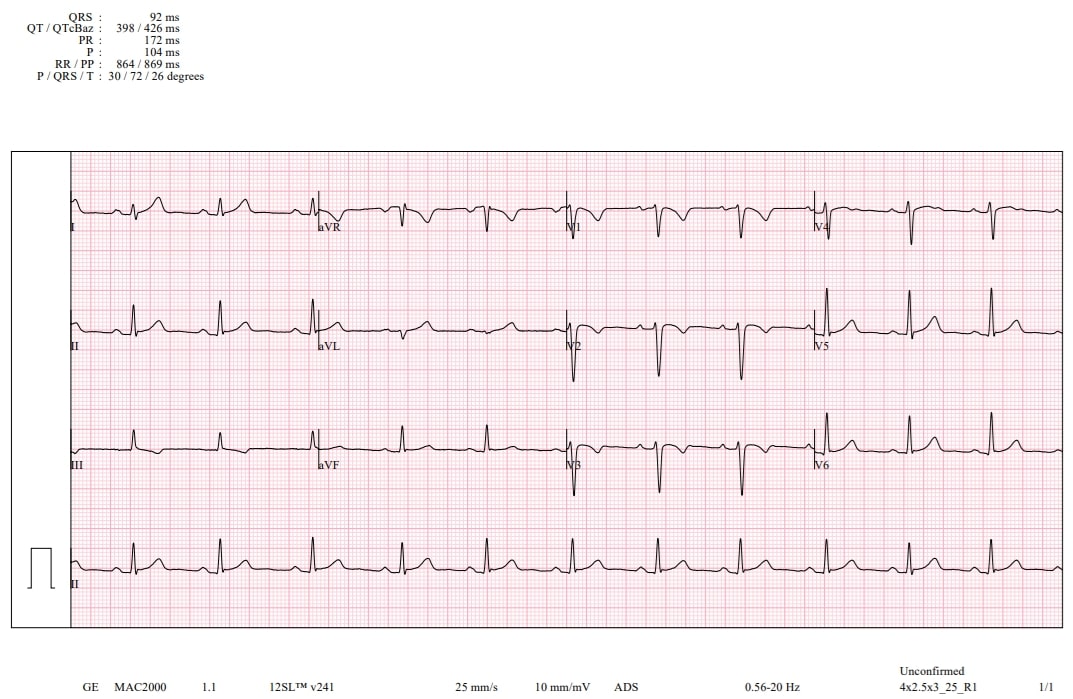


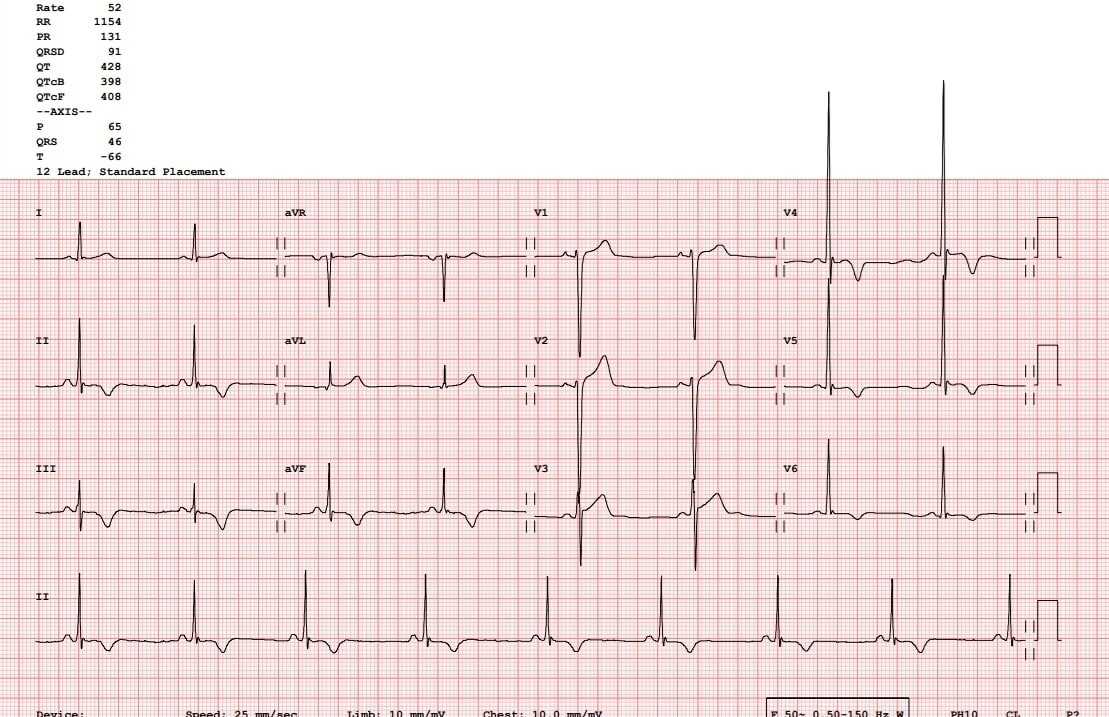


**Supplementary Figure S1**: ECGs of Anterior and Inferolateral T Wave Inversion Among Para-Football Players

**Reference**

1. Drezner JA, Sharma S, Baggish A, Papadakis M, Wilson MG, Prutkin JM, Gerche A La, Ackerman MJ, Borjesson M, Salerno JC, et al. International criteria for electrocardiographic interpretation in athletes: Consensus statement. *Br J Sports Med*. 2017;51:704.
